# MicroRNAs in the Regulation of Solute Carrier Proteins Behind Xenobiotic and Nutrient Transport in Cells

**DOI:** 10.3389/fmolb.2022.893846

**Published:** 2022-06-09

**Authors:** Colleen Yi, Ai-Ming Yu

**Affiliations:** Department of Biochemistry and Molecular Medicine, School of Medicine, University of California, Davis, Sacramento, CA, United States

**Keywords:** microRNA, nutrient, xenobiotic, therapy, cancer, solute carrier, regulation, therapy

## Abstract

Altered metabolism, such as aerobic glycolysis or the Warburg effect, has been recognized as characteristics of tumor cells for almost a century. Since then, there is accumulating evidence to demonstrate the metabolic reprogramming of tumor cells, addiction to excessive uptake and metabolism of key nutrients, to support rapid proliferation and invasion under tumor microenvironment. The solute carrier (SLC) superfamily transporters are responsible for influx or efflux of a wide variety of xenobiotic and metabolites that are needed for the cells to function, as well as some medications. To meet the increased demand for nutrients and energy, SLC transporters are frequently dysregulated in cancer cells. The SLCs responsible for the transport of key nutrients for cancer metabolism and energetics, such as glucose and amino acids, are of particular interest for their roles in tumor progression and metastasis. Meanwhile, rewired metabolism is accompanied by the dysregulation of microRNAs (miRNAs or miRs) that are small, noncoding RNAs governing posttranscriptional gene regulation. Studies have shown that many miRNAs directly regulate the expression of specific SLC transporters in normal or diseased cells. Changes of SLC transporter expression and function can subsequently alter the uptake of nutrients or therapeutics. Given the important role for miRNAs in regulating disease progression, there is growing interest in developing miRNA-based therapies, beyond serving as potential diagnostic or prognostic biomarkers. In this article, we discuss how miRNAs regulate the expression of SLC transporters and highlight potential influence on the supply of essential nutrients for cell metabolism and drug exposure toward desired efficacy.

## Introduction

The first microRNA (miRNA), namely lin-4, was originally discovered in *Caenorhabditis elegans* in 1993 (Lee et al., 1993; Wightman et al., 1993). It was found that lin-4 suppressed the translation of lin-14 through complementary base pairing, and lin-4 function is crucial for larval development (Lee et al., 1993; Wightman et al., 1993). Since then, functional miRNAs are revealed as a superfamily of noncoding RNAs (ncRNAs) in almost all species, including humans (Pasquinelli et al., 2000). About 18–25 nucleotides in length, genome-derived miRNAs generally act on the 3′-untranslated region (3′UTR) of target mRNAs to control posttranscriptional gene expression (Ambros, 2004; Bartel, 2004; Friedman et al., 2009). As a result, miRNAs are crucial regulators of essentially all cellular processes, and dysregulation of some miRNAs may be associated with specific diseases. Indeed, many miRNAs are involved in the regulation of cancer cell properties, including cell cycle, proliferation, apoptosis, metabolism, senescence, stemness, and immunity as well as xenobiotic drug metabolism and disposition (Yu and Pan, 2012; Yu et al., 2016), whereas some miRNAs are dysregulated in cancer cells (Anfossi et al., 2018; Dragomir et al., 2021). With an improved understanding of miRNA functions and cancer biology, new miRNA-based therapeutic strategies are emerging for the treatment of various types of cancer diseases (Rupaimoole and Slack, 2017; Yu A.-M. et al., 2020; Kara et al., 2022).

The dysregulated uptake and metabolism of key nutrients, such as glucose and amino acids (AAs), has been identified as a hallmark of cancer (Hanahan and Weinberg, 2011; Pavlova and Thompson, 2016). Among them, the influx and efflux of nutrients are mediated by solute carrier (SLC) transporters, and some SLCs have been characterized as either tumor suppressors or promoters with roles in many cellular processes (Rashid et al., 2021). SLCs are facilitative transporters or secondary active transporters but do not use ATP for energy as ATP-binding cassette (ABC) transporters do (Pizzagalli et al., 2021). In addition to essential nutrients, ions, and endobiotic metabolites (Zhang et al., 2019), some SLCs are also involved in the transport of therapeutics and their metabolites (Zhou et al., 2017). Meanwhile, some miRNAs are also revealed to modulate cancer cell metabolism (Pedroza-Torres et al., 2019) through the direct targeting of specific nutrient metabolic enzymes, in addition to a variety of SLC transporters. Interestingly, even though SLC proteins are the largest group of transporters, with over 450 members, SLCs are understudied considering their critical roles in the transport of essential nutrients and metabolites as well as medications and toxins pivotal to physiology, disease etiology, and pharmacotherapy (César-Razquin et al., 2015).

In this article, we will provide an overview of miRNA-controlled regulation of SLC family transporters involved in the transport and homeostasis of nutrients (e.g., carbohydrates and AAs) that are critical for cell survival with a focus on those related to cancer cell metabolism, as well as SLC drug transporters that are important for clinical therapy. How miRNA dysregulation can reprogram cancer cell metabolism will be highlighted. Furthermore, we will discuss the possibility and strategies for applying miRNA cancer biology, including miRNA–SLC interactions, to the development of new anticancer therapies.

## MicroRNA Biogenesis and Functions

**FIGURE 1:**
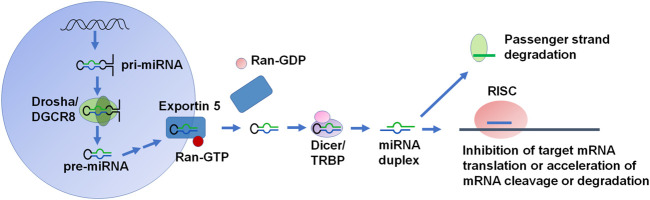
The canonical biogenesis and function of miRNAs in cells. Canonical miRNA biogenesis starts from the production of primary miRNA (pri-miRNA) transcript from DNA primarily by RNA polymerase II, which is processed subsequently by DROSHA to precursor miRNA (pre-miRNA) within the nucleus. Pre-miRNA is exported into the cytoplasm by Exportin 5. In the cytoplasm, the pre-miRNA detaches from Exportin 5/Ran-GDP and then cleaved by DICER to offer miRNA duplex. After the miRNA duplex is unwound, the passenger strand is degraded while the guide strand or mature miRNA is preferably loaded into the RISC. Functional miRNA within the RISC binds to the complementary MRE site in the 3′UTR of target transcript to inhibit mRNA translation or accelerate its cleavage or degradation to achieve posttranscriptional gene regulation.

The canonical miRNA biogenesis pathway begins with the transcription of the primary miRNA (pri-miRNA) by RNA polymerase II within the nucleus (Figure 1). DROSHA and the cofactor DiGeorge Syndrome Critical Region 8 (DGCR8) form microprocessor to cut the pri-miRNA into a shorter precursor miRNA (pre-miRNA) (Denli et al., 2004). Pre-miRNA is exported into the cytoplasm through binding with exportin 5 and Ran-GTP. The pre-miRNA is then cleaved by DICER to the miRNA duplex, in which the guide strand is loaded into the RNA-induced silencing complex (RISC) consisting of Argonaute (AGO) family proteins, and the passenger strand is degraded (Schwarz et al., 2003) (Figure 1). There are additional non-canonical miRNA biogenesis pathways, which can be described as DROSHA/DGCR8-independent and/or DICER-independent (O'Brien et al., 2018). The active miRNA within RISC thus acts on the 3′UTR of its target mRNA to inhibit translation or increase the cleavage and degradation of the mRNA to achieve posttranscriptional gene regulation (Figure 1). Interestingly, a single miRNA may target many transcripts (Brennecke et al., 2005) so that miRNA dysregulation can have widespread effects in diseased cells. Indeed, miRNAs are involved in the regulation of almost all cellular processes including cancer cell tumorigenesis, progression, and metastasis, in which miRNAs may act as either tumor suppressors or oncomiRs, due to the ability to regulate most protein-coding genes (Dragomir et al., 2021).

Multiple factors influence miRNA expression or function. Most miRNA coding genes are in fragile sites or cancer-associated regions (Calin et al., 2004). MiRNA may be dysregulated by multiple mechanisms including gene deletions, amplifications, and mutations as well as alterations of relevant transcription factors, binding proteins, epigenetics, and posttranscriptional modifications (Dragomir et al., 2021). Indeed, many miRNAs have been reported as downregulated in cancer cells (Lu et al., 2005). One relating factor is the changes in DROSHA or DICER levels in specific cancers (Hata and Kashima, 2016; Ali Syeda et al., 2020). Furthermore, circular RNAs (circRNAs) have been widely reported as miRNA sponges that may prevent miRNA binding to their mRNA targets (Yarmishyn et al., 2022). The dysregulation of miRNA has been proven to influence all hallmarks of cancer (Peng and Croce, 2016; Van Roosbroeck and Calin, 2017).

## Cancer Cell Metabolism and Roles of SLC Transporters

**FIGURE 2:**
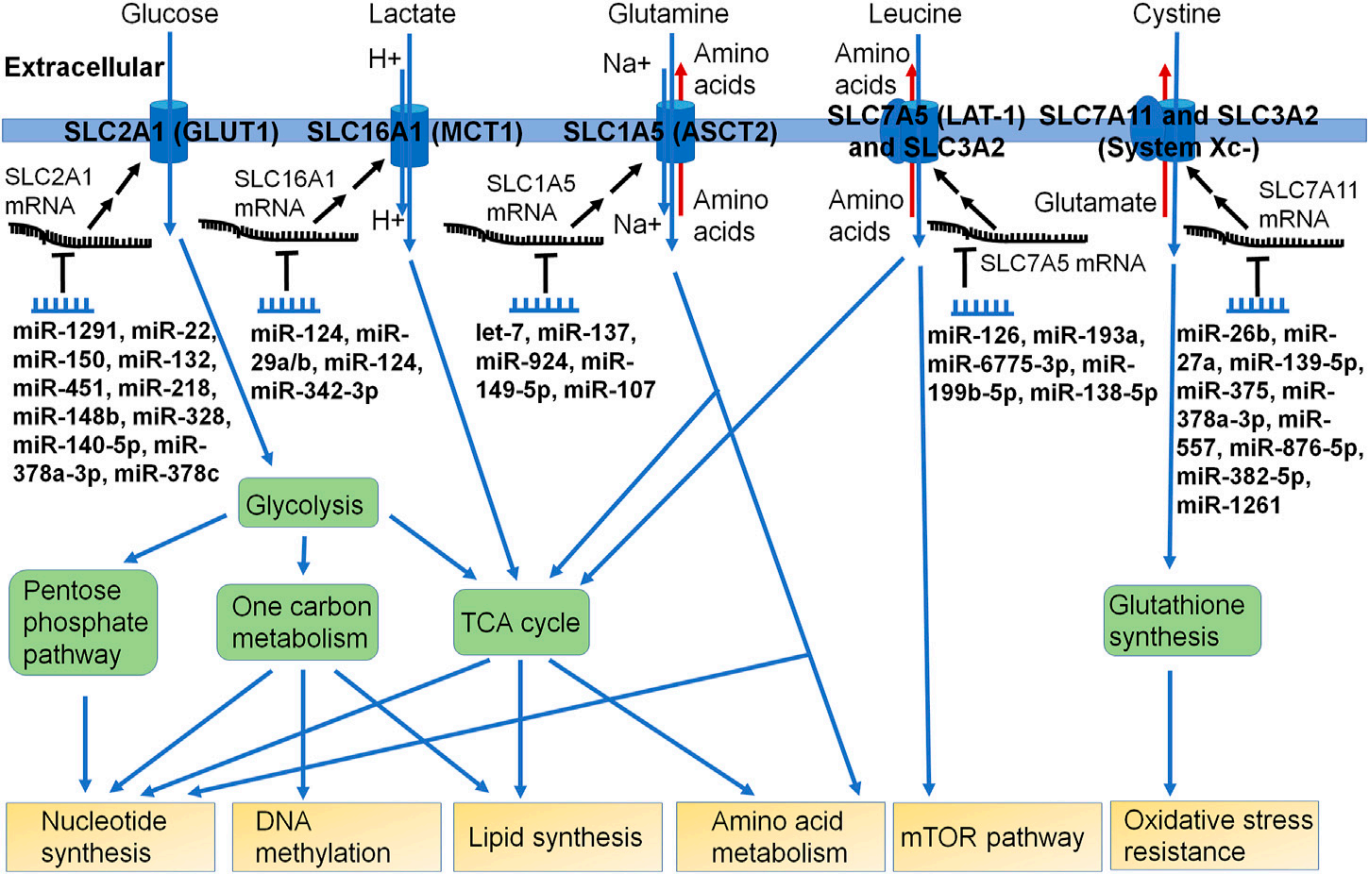
Many miRNAs have been shown to control the expression of specific SLC transporters critical for nutrient uptake or efflux in cells. The SLC transporters fuel multiple metabolic pathways, such as aminolyses, glycolysis, pentose phosphate pathway, one carbon metabolism, and TCA cycle. Some miRNAs have been identified to directly modulate the posttranscriptional gene expression of SLC transporters, which can impact the supply and homeostasis of nutrients or metabolic building blocks crucial for cell survival and growth. As certain SLC transporters are involved in the influx or efflux of xenobiotic drugs or toxins, the significance of the miRNA-controlled regulation of such SLCs in xenobiotic disposition and pharmacotherapy remains elusive.

Nutrients including carbohydrates (e.g., glucose), amino acids (e.g., glutamine), and vitamins (e.g., folate) are crucial for cell survival. The supply and metabolism of these nutrients (Figure 2) provide building blocks for other essential molecules (e.g., ATP and nucleotides) as well as providing reducing power from NADH, NADPH, and FADH_2_, which are dependent upon many respective SLC transporters and metabolic enzymes. Diseased cells such as carcinoma cells are faced with both a nutrient-poor environment and increased demands for growth and proliferation, which necessitates changes in gene regulation to reprogram nutrient metabolism to maintain necessary energetic and metabolite supplies (Pavlova and Thompson, 2016; Wei et al., 2020; Papalazarou and Maddocks, 2021).

The SLC superfamily encompasses over 450 proteins in humans, where proteins with at least 20% sequence similarity are grouped into a family (Hediger et al., 2013; Schumann et al., 2020). A list of SLCs and their characteristics, such as transport type, substrates, and localization, can be accessed through the Bioparadigms SLC Tables website (https://www.bioparadigms.org/slc/intro.htm), which has been validated by the HUGO Gene Nomenclature Committee (HGNC) (Hediger et al., 2013). Interestingly, some SLCs in different families lack sequence similarity but still have similar substrates. For instance, the SLC25 family members, which are present in both prokaryotes and eukaryotes, do not have significant sequence similarity to the SLC1A family; however, SLC25A22 and SLC1A1 are both glutamate transporters (Schlessinger et al., 2010). This similarity in function despite sequence differences is likely due to convergent evolution (Schlessinger et al., 2010). From evolutionary analysis based on the sequence, it has been found that 59% of human SLCs are also present in prokaryotes and the common ancestor of Eukaryotes, Eubacteria, and Archaea had SLCs (Hoglund et al., 2011).

SLCs are either passive facilitative transporters, which allows a substrate to move down its gradient, or secondary active transporters, where one substrate travels down its gradient to fuel the transport of another substrate against its gradient. Many transcription factors are known to regulate SLC levels in cancer; for instance, MYC and HIF1α are predicted to target SLC38A1 (Bröer et al., 2016; Panda et al., 2020). MiRNAs have important roles in posttranscriptional regulation, whose interactions with SLCs will be reviewed in further sections. Some common posttranslational modifications include N-glycosylation, palmitoylation, acetylation for control over mitochondrial SLC activity, phosphorylation to control transport activity, SUMOylation, and ubiquitination for degradation (Czuba et al., 2018). SLCs are predicted to have between 1 and 16 transmembrane domains based on hydropathy plots, and most known structures have transmembrane pseudosymmetry. Nevertheless, there are just a limited number of high-resolution structures due to challenges in purifying and analyzing transmembrane proteins (Bai et al., 2017; Pizzagalli et al., 2021). Over 80 SLCs have been associated with monogenic disorders, exemplifying their crucial role in human health and metabolism (Lin et al., 2015).

As revealed by Warburg (Warburg, 1956), there is an increase in glucose use and subsequent lactate production, known as aerobic glycolysis, in cancer cells even in the presence of oxygen. Many changes are linked to the Warburg effect, including an increase in the SLC2A family of glucose transporters, glycolytic enzymes, lactate dehydrogenase, SLC16A family of lactate transporters, and flow into the pentose-phosphate pathway (PPP) (Kozal et al., 2021). For example, elevated SLC2A1 (GLUT1) and SLC2A3 (GLUT3) have been associated with increased cancer metabolism (Ancey et al., 2018). The SLC16A family lactate transporters have also been reported to be dysregulated, in part to maintain intracellular pH for continued growth with increased lactate production (Felmlee et al., 2020).

The PPP consists of the non-oxidative and the oxidative branches. The non-oxidative branch produces ribose 5-phosphate, while the oxidative branch is necessary for NADPH and ribulose 5-phosphate production. Ribose 5-phosphate can then be used for nucleotide synthesis. Cancer cells with high SLC7A11 import more cystines, which is converted into cysteine with the use of NADPH for glutathione synthesis. This results in PPP dependency to maintain NADPH levels for redox homeostasis (Liu et al., 2020). Glucose starvation or SLC2A inhibition with KL-11743 results in cell death due to insufficient NADPH (Liu et al., 2020).

The metabolite 3-phosphoglyceric acid from glycolysis may be converted into serine for use in one-carbon metabolism (Figure 2). In HEK293T cells, half of the serine was shown to be synthesized from glucose (Locasale et al., 2011). Other possible sources of carbon for one-carbon metabolism include glycine and threonine (Locasale, 2013). One-carbon metabolism is made of the folate and methionine cycle. The folate cycle produces 10-formyl-THF, which can be used in purine synthesis. The methionine cycle produces S-adenosylmethionine, which can be used by methyltransferases. By altering the use of glucose in metabolic pathways, cancer cells can increase the production of building blocks that are not in as high of demand for cellular senescence.

The Warburg effect reduces glucose-derived pyruvate levels, so the TCA cycle also relies on glutaminolysis to supply alpha-ketoglutarate. In the TCA cycle, citrate can be converted into acetyl-CoA, which will serve as precursors for fatty acid synthesis (Figure 2). Malate can be exported into the cytoplasm to produce pyruvate and NADPH. Oxaloacetate can be converted into aspartate for nucleotide synthesis. Two important roles of glutamine are conversion into lactate for NADPH production necessary for fatty acid synthesis and TCA cycle anaplerosis (Deberardinis et al., 2007). SLC1A5 (ASCT2) is a highly studied transporter of glutamine (Bröer, 2020). After entry into the cell, glutamine can be converted into glutamate by glutaminase. Glutamate may then be converted into alpha-ketoglutarate by glutamate dehydrogenase or processed by transaminases, which will lead to the production of nonessential amino acids (Figure 2) (Yang et al., 2017).

SLC7A5 (LAT1) with SLC3A2 is another crucial amino acid transporter of essential amino acids, including leucine. Lysosomal-associated transmembrane protein 4b (LAPTM4b) has been shown to bind to SLC7A5/SLC3A2 to bring it to the lysosomal membrane, which is crucial for leucine to enter the lysosome to activate the mammalian target of rapamycin 1 (mTORC1) (Milkereit et al., 2015). Activation of mTORC1 in cancer cells results in tumor growth, survival, and metastasis by stimulating synthesis pathways such as for nucleotide, lipid, and protein (Hua et al., 2019). To maintain these metabolic processes, sufficient nutrients must be transported into the cells in which SLC transporters are largely involved (Figure 2).

## Carbohydrate Transporters and MicroRNA Regulation

The SLC2 (glucose transporter or GLUT) and SLC5 family proteins are critical for the transport of carbohydrates across the cell membrane (Papalazarou and Maddocks, 2021). For instance, a crucial role documented for transporters SLC2A1-4 or GLUT1-4 is to facilitate the passive transport of glucose. After import into the cell, glucose is phosphorylated by hexokinase into glucose 6-phosphate to discourage glucose efflux. SLC2A1-4 is upregulated in multiple cancer types (e.g., pancreatic, lung, and prostate), so there is significant interest in developing therapeutics targeting these transporters (Adekola et al., 2012; Holman, 2020; Pliszka and Szablewski, 2021). The SLC5 family are sodium symporters that can import their substrates, including glucose, against their gradient due to the Na^+^ electrochemical gradient. Members of the family, such as SLC5A2 and SLC5A5, are upregulated in certain cancer types (Gyimesi et al., 2020).

In concordance with the dysregulation of SLC2/5 family carbohydrate transporters and regulatory miRNAs in cancer cells, some miRNAs have been demonstrated to govern the expression of SLC2/5 genes (Table 1), in which the facilitative uniporter SLC2A1/GLUT1 is particularly well studied. SLC2A1 is present in most cells, with the highest expression in erythrocyte membranes (Mueckler and Thorens, 2013). Many SLC2A1 targeting miRNAs are downregulated in cancer cells, such as miR-148b in gastric cancer, miR-218 in bladder cancer, and miR-328 in colon cancer (Ding et al., 2017; Li et al., 2017; Santasusagna et al., 2018). Both miR-22 and miR-140-5p have been reported to target SLC2A1 in breast cancer (Chen B. et al., 2015; He et al., 2019). In prostate cancer, miR-378a and miR-132 can modulate SLC2A1 expression (Qu et al., 2016; Cannistraci et al., 2022). The oncogenic long ncRNA plasmacytoma variant translocation 1 was shown to sponge miR-378c in lung adenocarcinoma and prevent miR-378c binding to SLC2A1, resulting in an increase in SLC2A1 levels (Xia et al., 2021). MiR-378a-3p was found to be downregulated in prostate cancer, and miR-378a-3p was revealed to reduce SLC2A1 expression to interfere with glycolytic pathway and cell proliferation (Cannistraci et al., 2022).

**TABLE 1:** SLC2 and SLC5 family carbohydrate transporters regulated by miRNAs and the impact.

Transporters	Carbohydrate substrates	miRNAs	Diseases and/or model systems	Major findings	References
SLC2A1/GLUT1	D-glucose D-glucosamine	miR-1291-5p	Renal cell carcinoma cell lines A498 and 786-O and 27 pairs of clear cell renal cell carcinoma and noncancerous clinical specimens	Transfection with miR-1291 reduced SLC2A1 protein by about 30% inverse correlation between SLC2A1 and miR-1291 in clinical specimens	Yamasaki et al. (2013)
		miR-22-3p	Breast cancer MDA-MB-231 and MCF-7 cell lines 122 clinical breast cancer samples	Luciferase reporter assay with transfection of miR-22 and SLC2A1 3′UTR vector resulted in about 40% decrease of luciferase activity	Chen B. et al. (2015)
		miR-150-5p	Human peripheral blood samples Jurkat cell line	CD4^+^ T cells Overexpression of miR-150 decreased SLC2A1 mRNA and protein by about 50%	King et al. (2016)
		miR-132-3p	Prostate cancer cell lines PC-3, DU-145, and kidney cell line HEK293T GSE36802 prostate cancer dataset	miR-132 mimic transfection reduced SLC2A1 mRNA level by 50%–70%, while miR-132 inhibition increased SLC2A1 mRNA by about 3-fold	Qu et al. (2016)
		miR-218-5p	Bladder cancer cell lines T24 and EJ	miR-218 mimic transfection reduces SLC2A1 protein by 40%–50% SLC2A1 knockdown sensitizes cells to cisplatin	Li et al. (2017)
		miR-148b-3p	Clinical gastric cancer specimens and gastric cell lines BGC-823 and MKN45	Transfection with miR-148b significantly lowered SLC2A1 protein 72 h after transfection but not 48 h after transfection	Ding et al. (2017)
		miR-328-3p	Colon cancer cell lines, LOVO and SW480, 47 paired clinical colon cancer and normal tissue samples	Treatment with pre-miR-328 reduced SLC2A1 protein levels by about 20% in LOVO cells and about 60% in SW480 cells	Santasusagna et al. (2018)
		miR-140-5p	Breast cancer cell lines MCF-7, MDA-MB-231, T47D, BT549, SKBR3, MCF10A, and kidney cell line HEK293T TCGA datasets Female NOD-SCID mice	miR-140-5p overexpression reduces SLC2A1 mRNA by about 60%	He et al. (2019)
		miR-378a-3p	Prostate cancer cell lines 22Rv1, LNCaP, PC3, DU145 RWPE-1 and BPH-1 cell lines TCGA and MSKCC prostate cancer databases	Transfection of miR-378a downregulates SLC2A1 protein in both LNCaP and PC3 cells	Cannistraci et al. (2022)
		miR-378c-5p	Lung adenocarcinoma cell lines A549, NCI-H1975, Calu-3, and normal lung epithelial cell line BEAS-2B	Transfection with miR-378c mimic reduced SLC2A1 mRNA by about 75% and protein by about 50%. PVT1 sponges miR-378c to prevent binding to SLC2A1	Xia et al. (2021)
SLC2A3/GLUT3	D-glucose	miR-129-5p	Gastric cancer cell lines SGC-7901, MGC-803, and kidney cell line HEK 293T	Transfection with miR-129-5p mimics reduced SLC2A3 protein by 70%–90%	Chen et al. (2018)
		miR-29c-3p	Prostate cancer cell lines LNCaP, 22RV1, DU145, PC-3 and WPMY-1 GSE21036 database, 18 normal prostate samples, and 57 prostate cancer samples	Transfection with miR-29c mimics decreased SLC2A3 mRNA by about 50%	Li J. et al. (2018)
		miR-106a-5p	Glioblastoma cell lines U251 and LN229 3 normal brain samples, 6 grade II glioma samples, 6 grade III glioma samples, and 7 grade IV glioma samples	Luciferase reporter assay with transfection of miR-106a and SLC2A3 3′UTR vector resulted in about 50% decrease of luciferase activity	Dai et al. (2013)
		miR-195-5p	Bladder cancer cell lines T24 and HUC	Luciferase reporter assay with transfection of miR-195-5p and SLC2A3 3′UTR vector resulted in 57% decrease of luciferase activity	Fei et al. (2012)
		miR-184-3p	Clear cell renal cell carcinoma cell lines 769-P, Caki-1, 786-O, and normal kidney HK-2 cells Gene Expression Omnibus (GEO) database, GSE53757, and GSE53757 datasets Clear cell renal cell carcinoma clinical samples Xenograft mouse model	Negative correlation between miR-184 and SLC2A3 in clear cell renal cell carcinoma Long non-coding RNA LINC01094 sponges miR-184 to prevent binding to SLC2A3, with direct interactions confirmed by the dual-luciferase reporter assay	Xu et al. (2020)
SLC2A4/GLUT4	D-glucose D-glucosamine	miR-199a-5p	Type 2 diabetes 192 Han Chinese individuals HEK293T cells and Rat L6 muscle cells	Transfection with miR-199a mimic significantly downregulated SLC2A4 protein, while miR-199a inhibitor increased protein	Yan et al. (2014)
		miR-223-5p	Insulin-resistant adipose tissue 33 clinical samples	miR-223 overexpression does not downregulate SLC2A4 mRNA but does downregulate protein by about 30%	Chuang et al. (2015)
		miR-106b-5p	Insulin resistance male Wistar rats, L6 skeletal muscle cells	About 70% reduction in SLC2A4 protein level with miR-106b mimic transfection	Zhou et al. (2016)
		miR-17-5p	Insulin-resistant skeletal muscle L6 cell line, Wistar rats	Negative correlation of miR-17 and SLC2A4	Xiao et al. (2018)
SLC5A3	Na^+^ Myo-inositol	miR-204-5p	Parkinson’s disease Neuroblastoma cell lines SK-N-SH and SK-N-AS	Transfection with miR-204-5p reduced SLC5A3 by about 70%. Long non-coding RNA NORAD sponges miR-204-5p to prevent binding to SLC5A3	Zhou et al. (2020)

## Lactate Transporters and MicroRNA Regulation

The SLC16A subfamily proteins are monocarboxylate transporters (MCTs) that use the proton gradient to transport endobiotic (e.g., lactate and pyruvate) and xenobiotic (e.g., γ-hydroxybutyric acid) (Morris and Felmlee, 2008). Of particular interest are SLC16A1 (MCT1), which is present in the plasma membrane of most cells, and SLC16A3 (MCT4), which is more highly expressed in glycolytic cells such as skeletal muscle cells (Price et al., 1998; Payen et al., 2020). SLC16A1 may also be expressed in the nuclear, sarcolemma, and mitochondrial membrane (Park et al., 2018). SLC16A1 and SLC16A3 are overexpressed and associated with poor prognosis in cancers, such as pancreatic cancer (Yu S. et al., 2020). Lactate can be used by oxidative cancer cells as a signaling molecule with dysregulated SLC16A proteins (Payen et al., 2020). During metabolic symbiosis, SLC16A1 is involved in the influx of lactic acid into oxidative cancer cells, while SLC16A3 is involved in the efflux of lactate from glycolytic cancer cells (Sonveaux et al., 2008; Payen et al., 2020). SLC16A1 inhibition blocks tumor growth by increasing glucose use by oxidative cancer cells, which results in hypoxic cancer cell death due to glucose starvation (Sonveaux et al., 2008; Wang et al., 2022). Additionally, high levels of extracellular lactate will inhibit lactate efflux, which can block T-lymphocytes from proliferating and contribute to immune resistance (Fischer et al., 2007).

Some miRNAs have shown to modulate SLC16A1 expression and influence lactate transport in cancer cells (Table 2). In medulloblastomas, miR-124 was found to directly regulate SLC16A1, but miR-124 was downregulated in most tumors, which lead to an increase in SLC16A1 (Li et al., 2009). In triple negative breast cancer, miR-342-3p was found to directly reduce SLC16A1; as miR-342-3p was downregulated due to a lack of estrogen receptor, SLC16A1 levels were shown to be increased (Romero-Cordoba et al., 2018). Transfection with miR-342-3p mimic reduced SLC16A1 protein levels in breast cancer cells, and miR-342-3p overexpression reduced lactate influx in MDAMB468 cells, resulting in the accumulation of extracellular lactate, which disrupted metabolic symbiosis and increased competition for glucose (Romero-Cordoba et al., 2018) (Table 2).

**TABLE 2:** Multiple miRNAs are shown to regulate SLC16A1/MCT1.

Transporters	Endogenous substrates	miRNAs	Diseases and/or model systems	Observations	References
SLC16A1/MCT1	L-lactic acid Pyruvic acid β-D-hydroxybutyric acid	miR-124-3p	Medulloblastoma cell lines D283, D341, D384, D425, D458, DAOY, and ONS-76 cells	Transfection with miR-124 mimics reduced SLC16A1 protein by 70% in DAOY cells and over 90% in the other medulloblastoma cell lines	Li et al. (2009)
			29 clinical medulloblastoma samples		
		miR-29a-3p, miR-29b-3p, and miR-124-3p	Pancreatic beta cell line MIN6, hepatoma cell line mhAT3F, and HEK293 cell lines	miR-29b overexpression significantly downregulated SLC16A1 protein, while miR-29a and miR-124 overexpression almost eliminated SLC16A1 protein expression	Pullen et al. (2011)
			C57BL/6 mice		
		miR-342-3p	Triple negative breast cancer	miR-342-3p overexpression caused SLC16A1 protein levels to be 50% of the control in MDAMB468 cells, 64% in BT549 cells, 38% in Sum149 cells, 73% in SUM159, but did not cause downregulation in MDAMB157 cells	Romero-Cordoba et al. (2018)
			Breast cancer cell lines MDAMB468, BT549, SUM159, MDAMB157 cell lines		
			164 clinical breast cancer samples		
			TCGA and METABRIC data sets		

## Amino Acid Transporters and MicroRNA Regulation

### SLC1A5

SLC1A5 (alanine, serine, cysteine transporter 2 or ASCT2) is a Na^+^-dependent antiporter in the plasma membrane involved in the exchange of neutral amino acids, such as glutamine. It has been known since the 1950s that HeLa cells use significantly more glutamine than other AAs (Eagle, 1955). Once imported into the cell, glutamine is converted to glutamate, which encourages the continued influx of glutamine and prevents efflux. SLC1A5 is commonly expressed in lung, skeletal muscle, large intestine, kidney, and adipose tissue (Kanai et al., 2013). SLC1A5 levels are increased in tissues that need high levels of glutamine for metabolism, as well as in cancer cells, including cancer from tissues that normally lack SLC1A5 (Lopes et al., 2021).

MiR-137 has been shown to directly bind to SLC1A5 in melanoma, which suppresses ferroptosis through the downregulation of SLC1A5 (Luo et al., 2018) (Table 3). MiR-137 is commonly downregulated in melanoma, so these cells might be sensitive to ferroptosis (Luo et al., 2018). Indeed, miR-137 was shown to regulate apoptosis, autophagy, and ferroptosis (Luo et al., 2018). In addition, miR-107, miR-149-5p, and miR-924 were shown to regulate SLC1A5 in esophageal cancer, breast cancer, and non-small-cell lung cancer cells respectively; however, they were sponged by circular RNA to prevent downregulation of SLC1A5 (Chang et al., 2021; Wang J. et al., 2021; Liu et al., 2022) (Table 3). There is also an interest in combining the inhibition of SLC1A5 and SLC7A5, since both are involved in the transport of crucial AAs (Kanai, 2022).

**TABLE 3:** Specific miRNAs have been revealed to control the expression of amino acid transporters.

Transporters	Endogenous substrates	miRNAs	Diseases and/or model systems	Findings	References
SLC1A1/EAAT3	L-glutamic acid L-aspartic acid L-cysteine	miR-26a-5p	20 clinical multiple sclerosis samples Oligodendrocyte hybrid cell line MO3-13	Transfection of miR-26a in MO3-13 cells reduced SLC1A1 mRNA by 50% and protein 30%. In 20 patients with MS treated with INF-β, miR-26a levels were increased SLC1A1 mRNA was decreased	Potenza et al. (2018)
		miR-183-5p, miR-96-5p, miR-182-5p	Light adaptation in mouse retina Mouse embryonic fibroblast NIH 3T3 cells C57BL/6 mice, Sprague-Dawley rats, and Wistar rats	miR-183/96/182 are upregulated in light-adapted mouse retinas and downregulated with dark adaptation Enhanced green fluorescent protein and firefly luciferase reporters to confirm miR-183/96/182 direct binding to SLC1A1	Krol et al. (2010)
		miR-96-5p	Multiple system atrophy Human cases from University of California San Diego Alzheimer Disease Research Center 5 transgenic mouse models (MBP1-hαsyn tg, MBP29-hαsyn tg, mThy1-hαsyn tg, mThy1-hAPP tg, mThy1-htau) and non-transgenic mice	Luciferase assay to confirm direct binding of miR-96 to SLC1A1	Ubhi et al. (2014)
		miR-96-5p	Neuroprotection against oxidative stress Neuroblastoma cell line SH-SY5Y and HEK293 cell line male ddY mice	Transfection with miR-96-5p downregulates SLC1A1 protein by about 40%	Kinoshita et al. (2014)
		Rno-miR-101b-3p and hsa-miR-101-3p	Rat model of major depressive disorder (FSL and FRL)	Luciferase reporter assay was used to confirm the binding of miR-101 in HEK 293 cells. miR101-b was decreased, while SLC1A1 mRNA levels were increased in in FSL mice	Wei et al. (2016)
SLC1A2/EAAT2	L-glutamic acid L-aspartic acid	miR-31-5p miR-200c-3p	Liver aging markers in liver transplants human liver biopsies HEK 293 cells	Transfection with miR-200c mimic reduced SLC1A2 mRNA by about 50%, while miR-31 mimic reduced SLC1A2 mRNA by over 90%	Capri et al. (2017)
SLC1A3/EAAT1/GLAST	L-glutamic acid L-aspartic acid	miR-155-5p	astrocytes in epilepsy Wistar rats	Luciferase reporter assay with transfection of miR-155-5p and SLC1A3 3′UTR vector resulted in about 60% decrease of luciferase activity	Gao et al. (2017)
SLC1A5/ASCT2	L-alanine, L-serine, L-cysteine, L-threonine, L-glutamine, L-asparagine, etc.	miR-137-3p	Melanoma cell lines A375 and G-361 and xenograft mouse model	miR-137 overexpression reduced SLC1A5 mRNA by about 50% and protein by about 75% miR-137 inhibition increased SLC1A5 mRNA about 1.5-fold and protein 1.5–1.6-fold. Transfection with miR-137 reduces SLC1A5 and blocks ferroptosis	Luo et al. (2018)
		miR-924-5p	Non-small-cell lung cancer cell lines NCI-H1299, HCC827, A549, H460, 16HBE cell lines and xenograft mouse model	Luciferase reporter assay with transfection of miR-924 and SLC1A5 3′UTR vector resulted in about 50% decrease of luciferase activity. Circ_0000463 is overexpressed in NSCLC and sponges miR-924 to prevent binding to SLC1A5	Liu et al. (2022)
		miR-149-5p	Breast cancer cell lines MDA-MB-231, BT-549, MCF-10A, and kidney cell line 293T 60 clinical paired breast cancer and normal tissue samples BALB/C nude mice	Luciferase reporter assay with transfection of miR-149-5p and SLC1A5 3′UTR vector resulted in about 60% decrease of luciferase activity. circular RNA septin 9 sponges miR-149-5p	Wang J. et al. (2021)
		miR-107-3p	Esophageal carcinoma cell lines ECA109, KYSE410, KYSE150, TE1, and esophageal epithelial cell line HEEC 39 clinical paired esophageal cancer and normal tissue samples Male xenograft mouse model	miR-107 overexpression decreased SLC1A5 protein by about 50%, while miR-107 inhibition increased protein by about 2-fold Circ-SFMBT2 sponges miR-107	Chang et al. (2021)
SLC7A1/CAT1	L-arginine, L-lysine, L-ornithine, L-histidine	miR-122-5p	Hepatocyte cell lines Huh7, HepG2, AML-12, WC-3, and kidney cell line 293T mouse samples and human primary hepatocytes	Negative correlation between miR-122 and SLC7A1 in primary human hepatocytes, Huh7, HepG2, and 293T, but not in AML-12	Chang et al. (2004)
			Cellular stress HeLa, HEK293, HepG2, Huh7, and DLD-1	miR-122 binds to SLC7A1 to repress translation, but HuR prevents repression during cellular stress	Bhattacharyya et al. (2006)
		miR-145-5p	10 male spontaneously hypertensive rats and 10 age-matched normotensive male Wistar-Kyoto rats	Inhibiting miR-145 resulted in about 2.5-fold increase in SLC7A1 mRNA	Wang and Jin, (2018)
SLC7A5/LAT1	Neutral AAs	miR-126-3p	Small cell lung cancer cell lines H69 and HTB-172 Primary small cell lung cancer samples	miR-126 overexpression reduced SLC7A5 mRNA more than 50%	Miko et al. (2011), Li H. et al. (2018)
			Gastric cancer cell lines SGC-7901, MKN-45, MKN-28, GES-1 cell lines 54 matched primary gastric cancer and noncancerous tissue samples	miR-126 expression downregulated SLC7A5 by about 50%	Wang et al., (2015)
			80 paired lung cancer and non-tumor samples Lung cancer cell lines A549, H226, H1299, and H446, and lung epithelial cell line BESA-2B	miR-126 inhibition increases SLC7A5 expression PVT1-5 sponges miR-126 to prevent SLC7A5 repression	Li H. et al. (2018)
		miR-193a-3p	XB130 effect on cancer cells WRO and MRO cells	miR-193a mimic reduced SCL7A5 protein levels by about 50% XB130 suppresses miR-193a	Takeshita et al. (2013)
		miR-6775-3p	Esophageal squamous cell carcinoma cell lines TE1, Eca109, Ec9706, and KYSE30 138 clinical esophageal squamous cell carcinoma clinical samples Xenograft tumor mouse model	In TE1 cells transfected with miR-6775-3p mimics, SLC7A5 was decreased by about 70%. In Ec9706 cells transfected with miR-6775-3p inhibitor, SLC7A5 was increased by about 2.5-fold	Meng et al. (2018)
		miR-328-3p	Human osteosarcoma 143B and MG63 cells	MiR-328-3p reduced SLC7A5 3′UTR luciferase reporter activities and protein outcomes but did not alter the overall homeostasis of AAs. Combination treatment with miR-328 plus cisplatin or doxorubicin synergistically inhibited osteosarcoma cell proliferation	Yi et al. (2020)
		miR-199b-5p	Endometrial adenocarcinoma cell lines HEC1A, Ishikawa, and hEEC 46 clinical endometrial cancer and 46 healthy tissue samples xenograft mouse model GSE115810 and GSE36389 datasets	Transfection with miR-199b-5p mimics decreased SLC7A5 protein by about 50%	Shu et al. (2021)
		miR-138-5p	Retinoblastoma cell lines WERI-RB-1, Y79, HXO-RB44, SO-RB50, and rectal adenocarcinoma cell line HRA, 33 retinoblastoma samples and 21 normal samples Mouse tumor model	Transfection with miR-138-5p reduced SLC7A5 protein by about 50% circ-FAM158A sponges SLC7A5	Zheng et al. (2021)
SLC7A11/xCT	Cystine and glutamate	miR-26b-5p	Breast cancer cell lines MCF7, HCC 1937, MDA-MB-231, and CCD-1095Sk	miR-26b overexpression reduces SLC7A11 protein, while miR-26b inhibitors increase SLC7A11 protein	Liu et al. (2011)
		miR-27a-3p	cisplatin resistance in bladder cancer Bladder cancer cell lines EJ/T24 and RT112 354 clinical bladder cancer samples	Transfection of cisplatin-resistant cells with pre-miR precursor to miRNA-27a decreased SLC7A11 protein and increased cisplatin sensitivity 100-fold	Drayton et al. (2014)
			Lung cancer cell line A549 and lung epithelial cell line Beas-2B GSE27262, GSE102287, GSE116959, GSE118370, and GSE19945 datasets 90 clinical paired NSCLC and normal tissue samples	Non-small-cell lung cancer Transfection with miR-27a-3p mimic reduced SLC7A11 expression by about 50%	Lu et al. (2021)
		miR-139-5p	Pancreatic carcinoma cell lines PANC-1, Bx-PC3, and HPDE TCGA-PAAD database 45 paired pancreatic carcinoma and normal tissue samples Xenograft mouse model	Transfection with miR-139-5p mimics reduced SLC7A11 mRNA by about 50%	Zhu et al. (2020)
		miR-375-3p	Oral squamous cell carcinoma cell lines Fadu, SCC-25, CAL-27, Tca8113, and Hs 680.Tg 40 clinical oral squamous cell carcinoma and paired normal samples	Transfection with miR-375 mimics reduces SLC7A11 by about 60%	Wu et al. (2017)
		miR-378a-3p	ferroptosis in I/R-induced renal injury Human kidney cell lines HK-2 and TCMK-1 I/R-induced kidney injury rats	miR-378a-3p mimic transfection does not have a significant impact on SLC7A11 mRNA but does reduce SLC7A11 protein by about 60%	Ding et al. (2020)
		miR-557-3p	Pancreatic cancer cell lines HPDE, Hs 766T, and SW1990 93 clinical paired pancreatic cancer and normal samples Xenograft mouse model	Transfection with miR-557 mimic reduced SLC7A11 protein by about 60%	Zhang T. et al. (2021)
		miR-876-5p	Oral squamous cell carcinoma cell lines Cal-27, SCC9, HOK, and kidney cell line 293T 34 oral squamous cell carcinoma tissues and 19 non-tumor samples male BALB/c nude mice	Transfecting cells with miR-876-5p inhibitor increases SLC7A11 by about 1.5-2-fold. CircCDR1 acted as an oncogene by sponging miR-876-5p	Cui et al. (Online ahead of print)
		miR-382-5p	Lidocaine-induced ferroptosis in ovarian and breast cancer Ovarian adenocarcinoma cell line SKOV-3 and breast cancer cell line T47D 38 clinical ovarian cancer samples and 50 clinical breast cancer samples Xenograft model with male mice	Transfection with miR-382-5p mimic reduces SLC7A11 mRNA by about 75%	Sun et al. (2021)
		miR-1261-5p	Hepatocellular carcinoma cell lines HepG2, BEL-7402, MHCC-97H, and hepatocyte cell line LO2 Female mouse xenograft model	Transfection with miR-1261 mimic reduced SLC7A11 expression by about 40–50% circ0097009 acts as a sponge for miR-1261	Lyu et al. (2021)
SLC25A12	Aspartate Glutamate	miR-302b-3p	MAVS-mediated antiviral innate immunity HEK293, HeLa, A549, MRC-5, HAP-1 cell lines	Transfection with miR-302b mimics reduced SLC25A12 by about 50%	Yasukawa et al. (2020)
SLC38A1/SNAT1	Neutral AAs	miR-593-3p	Insulin-promoted glucose consumption Hepatoma cell lines HepG2, BeL7402, and mouse myoblast cell line C2C12 41 paired liver cancer and normal samples Primary human hepatocyte cultures	Transfection with miR-593-3p mimics reduced SLC38A1 protein by about 60% and miR-593-3p inhibitor increased protein level by about 3-fold	Yang et al. (2016)
		MiR-138-5p	Colorectal cancer cell lines SW480 and SW620, normal colon cell line HCoEpiC 30 paired clinical colorectal cancer and normal samples Xenograft mouse model	Transfection with miR-138 reduces SLC38A1 mRNA by about 50%, while SLC38A1 protein is reduced by about 60% in SW480 cells and 50% in SW620 cells. The long noncoding RNA NEAT1 sponges miR-138	Wang S. et al. (2021)
		miR-150-5p	Pulmonary fibrosis Lung fibroblast cell line HFL1 Male Sprague-Dawley rats	Transfection with miR-150-5p mimic downregulated SLC38A1 by about 50%. lncRNA ZFAS1 sponges miR-150-5p	Yang et al. (2020)
		miR-511-3p	Hepatocellular carcinoma cell lines HepG2, Huh7, and L-02 TCGA data	Inhibition of miR-511 increases SLC38A1 mRNA by about 50% and increases protein by about 1.5–2-fold. LINC01559 sponges miR-511	Su and Wang, (2021)
		miR-485-5p	Colorectal cancer cell lines SW480, and SW620, and NCM460 31 paired clinical colorectal cancer and normal samples Male xenograft mouse model	Transfection with miR-485-5p decreased SLC38A1 protein by about 50%, while miR-485-5p inhibition increased protein by about 3-fold. CircRUNX1 sponges miR-485-5p	Yu et al. (2021)

### SLC7A5

SLC7A5 (L-type amino acid transporter 1 or LAT1) forms heterodimer with SLC3A2 to function as a Na^+^ independent, large neutral amino acid antiporter (Kanai et al., 1998; Mastroberardino et al., 1998) that is commonly overexpressed in cancer cells (Kanai, 2022). SLC7A5/SLC3A2 is normally expressed in the plasma membrane of testis, bone marrow, brain, and placenta (Scalise et al., 2018). SLC7A5 was also found to be expressed in the cytoplasm of liver and skeletal muscle, as well as in the lysosomal membrane for mTORC1 activation (Milkereit et al., 2015; Kanai, 2022). SLC7A5-mediated import of leucine, as well as other essential AAs, is crucial for cancer cells. It has been proposed in the past that the glutamine imported through SLC1A5 may be used for SLC7A5 efflux; however, this has been challenged due to the low affinity of SLC7A5 for glutamine (Scalise et al., 2017).

MiR-126 has been identified as a direct regulator of SLC7A5 in both gastric and lung cancer, while miR-126 is downregulated in gastric cancer and can function as a tumor suppressor (Feng et al., 2010) (Table 3). While SLC7A5 is upregulated in gastric cancer cells, miR-126 directly targets SLC7A5 and miR-126 overexpression can inhibit cancer cell proliferation (Wang et al., 2015). Similarly, miR-126 is downregulated in small cell lung cancer, and miR-126 directly regulates SLC7A5 expression (Miko et al., 2011). Long ncRNA plasmacytoma variant translocation 1–5 has been shown to act as a sponge for miR-126 in lung cancer cell lines, which prevents miR-126 from regulating SLC7A5 (Li H. et al., 2018). In esophageal squamous cell carcinoma, elevated miR-6775-3p levels were associated with better prognosis, and overexpression of miR-6775-3p blocked tumor growth and metastasis in xenograft mouse model (Meng et al., 2018). It was also found that p53 directly upregulates miR-6775-3p and SLC7A5, then miR-6775-3p downregulates SLC7A5 as well as the MAGE-A family (Meng et al., 2018). In addition, SLC7A5 has been verified as a direct target for miR-328-3p, whereas the reduction of LAT1 protein levels by miR-328-3p in osteosarcoma cells does not alter the overall levels of AAs (Yi et al., 2020), highlighting the importance to examine the impact on substrate transport or balance and indicating the presence of complex regulatory network behind AA homeostasis. Nevertheless, miR-328-3p also modulates the expression of other cancer-related genes, including SLC2A1, and synergistically inhibits cancer cell proliferation with chemotherapeutics (Yi et al., 2020) (Table 3).

### SLC7A11

System Xc^−^ is a heterodimeric cystine/glutamate antiporter made of the subunits SLC7A11 and SLC3A2, which is normally primarily expressed in the plasma membrane of the central nervous system (Lewerenz et al., 2013). System Xc^−^ is upregulated in cancer cells to increase the production of glutathione from cystine, resulting in the prevention of ferroptosis (Liu et al., 2021). Without glutathione, glutathione peroxidase 4 is inactive, which causes an increase in intracellular lipid peroxidation, eventually culminating in ferroptosis (Han et al., 2020).

Multiple miRNAs have been identified as direct regulators of SLC7A11 (Table 3). MiR-375 is downregulated in oral squamous cell carcinoma, and treatment with miR-375 mimics reduced SLC7A11 levels and acted as a tumor suppressor (Wu et al., 2017). Similarly, miR-139-5p is reduced in pancreatic carcinoma, and the overexpression of miR-139-5p reduces SLC7A11 expression and also suppresses phosphatidylinositol 3-kinase and phosphorylated protein kinase B (Zhu et al., 2020). MiR-26b is downregulated in breast cancer cells, and treatment with miR-26b mimics downregulates SLC7A11 and causes apoptosis (Liu et al., 2011). MiR-27a was found to be downregulated in cisplatin-resistant bladder cancer cells, and overexpression of miR-27a repressed SLC7A11 expression and increased cell sensitivity to cisplatin (Drayton et al., 2014). MiR-382-5p was found to directly interact with SLC7A11 in ovarian and breast cancer cells, whereas miR-382-5p was downregulated and SLC7A11 was upregulated (Sun et al., 2021). Interestingly, treatment with lidocaine increased miR-382-5p, which resulted in ferroptosis due to a decrease in SLC7A11 (Sun et al., 2021). Some circular RNAs have also been found to sponge miRNAs to prevent their regulation of SLC7A11 in carcinoma cells. MiR-557 was sponged by circular RNA eukaryotic translation initiation factor 6 (circEIF6) in pancreatic cancer cells, miR-876-5p was sponged by circRNA CDR1 antisense RNA (circCDR1as) in oral squamous cell carcinoma cells, and miR-1261 was sponged by the circular RNA circ0097009 in hepatocellular cancer cells, all of which were linked to the upregulation of SLC7A11 (Cui et al., 2021; Lyu et al., 2021; Zhang T. et al., 2021) (Table 3).

## Drug-Transporting SLCs and MicroRNA Regulation

Some SLC transporters, such as organic anion- or cation-transporting polypeptides (OATs, OATPs, or OCTs), are crucial for the uptake or efflux of xenobiotic medications (e.g., statins and chemotherapeutics) and toxins (e.g., microcystin and phalloidin), besides endobiotic metabolites (Roth et al., 2012; Zhou et al., 2017; Brecht et al., 2020). For instance, the SLC28 (concentrative nucleoside transporters or CNT) and SLC29 (equilibrative nucleoside transporters or ENT) families are involved in the influx of nucleoside and nucleobase drugs, such as clofarabine and gemcitabine (Young et al., 2013). The SLCO (OATP), SLC22A (OCT OAT, etc.), and SLC15A (peptide transporter or PEPT) families are key for the uptake of some statins (e.g., pravastatin and pitavastatin) and anticancer drugs (e.g., paclitaxel and doxorubicin), which may have significant impact on pharmacokinetics (Zhou et al., 2017; Brecht et al., 2020). Among them, SLCO1B3 is present in the basolateral membrane of hepatocytes and transports the chemotherapeutics docetaxel, paclitaxel, doxorubicin, cisplatin, carboplatin, and oxaliplatin (Schulte and Ho, 2019).

Dysregulation of both miRNA and solute carrier transporters have been shown to be associated with chemoresistance (Si et al., 2019; Sun et al., 2020; Rashid et al., 2021). MiR-579-3p was found to directly downregulate SLCO1B3 in pancreatic cancer cells, whereas the androgen biosynthesis inhibitor abiraterone downregulates miR-579-3p (Barbier et al., 2021) (Table 4). SLCO1B3 is also involved in the influx of testosterone, so the increase in SLCO1B3 is associated with resistance to androgen deprivation therapy (Barbier et al., 2021). In acute lymphoblastic leukemia, miR-595 downregulates the methotrexate transporter SLC19A1 with rs1051296 G > T polymorphism, and this decrease in SLC19A1 causes reduced methotrexate influx and sensitivity (Wang et al., 2018). This study shows that a single nucleotide polymorphism may alter the miRNA regulation of a key chemotherapeutic transporter; however, this was tested in a single cell line, so validation remains to be done (Wang et al., 2018). Further experimental determination and validation are required to delineate the influence of the miRNA-controlled regulation of SLC transporters on drug exposure and therapeutic outcomes.

**TABLE 4:** MiRNAs regulate SLC15, SLC 19, SLC22, and SLCO family transporters.

Transporters	Substrates	miRNAs	Models	Findings	References
SLC15A1/PEPT1	Di-/tri-peptides 5-aminolevulinic acid	miR-92b-3p	Intestinal epithelial cells Colorectal adenocarcinoma cell line Caco2-BBE	Transfection with miR-92b precursor downregulates SLC15A1 mRNA by about 40%	Dalmasso et al. (2011)

		miR-193-3p	Colorectal adenocarcinoma cell lines Caco2, HT29 cells, and kidney 293T cells Clinical ulcerative colitis and normal samples female C57BL/6 mice, dnTGFβRII mice, and C57BL/6 mice	Transfection of miR-193a-3p mimic downregulated SLC15A1 protein by about 80%, while miR-193a-3p inhibitor increased SlC15A1 levels by about 5-fold	Dai et al. (2015)
SLC19A1/RFC1 with rs1051296 G > T polymorphism	Folate Methotrexate	miR-595-3p	Lymphoblastic leukemia cell line CEM-C1	Transfection with miR-595 mimic reduced SLC19A1 protein by about 50%	Wang et al. (2018)
SLC22A7/OAT2	Prostaglandin E2 Aminohippuric acid	miR-29a-3p	Hepatocellular carcinoma cell line HepG2, hepatic cell line HepaRG, and kidney 293T cells	Transfection with miR-29a-3p mimic reduced SLC22A7 mRNA by 63% and protein by 66%	Yu et al. (2015)
SLC22A18/ORCTL2	Organic cations	miR-137-3p	Non-small-cell lung cancer cell lines H522 and H23 100 non-small-cell lung cancer and paracancerous samples	Transfection with miR-137 mimics decreased SLC22A18 protein level by about 60%	Zhang et al. (2015)
SLCO4A1	Organic anion	miR-150-3p	Colon cancer cell line HCT116 and colon cell line NCM460 Xenograft mouse model	Transfection with miR-150-3p reduced SLCO4A1 mRNA by about 50% and protein by about 40%. LncRNA SLCO4A1-AS1 sponges miR-150-3p	Wu et al. (2021)
SLCO1B3	Organic anions Androgens	miR-579-3p	Prostate cancer cell lines 22Rv1, LNCaP, and VCAP cell lines	miR-579-3p downregulated SLCO1B3 and abiraterone reduced miR-579-3p expression	Barbier et al. (2021)

## Other SLC Transporters Regulated by MicroRNAs

Some miRNAs have been revealed to regulate key vitamin- and neurotransmitter-transporting SLCs in models outside of cancer (Table 5). MiR-103a was found to directly downregulate the ascorbic acid transporter SLC23A1, and treatment with miR-103a decreased the influx of ascorbic acid, also known as vitamin C, in the intestinal epithelial cell line Caco-2 (Subramanian et al., 2019). Additionally, miR-141 and miR-200a directly target the ascorbic acid transporter SLC23A2 in mouse bone marrow stromal cells, which reduced osteogenic differentiation (Sangani et al., 2015). In another study that used Caco-2 and HuTu-80 cells as well as mouse intestinal enteroids, miR-423-5p mimic downregulates the riboflavin transporter SLC52A3 and causes a decrease in riboflavin influx (Lakhan et al., 2017). Mouse mmu-miR-16-5p was found to directly downregulate the serotonin transporter SLC6A4, and the antidepressant fluoxetine, a selective serotonin reuptake inhibitor, was shown to upregulate mmu-miR-16-5p (Baudry et al., 2010). This suggests that one mechanism of decreasing serotonin influx by fluoxetine is through the miR-16-5p-mediated downregulation of serotonin transporter (Baudry et al., 2010).

**TABLE 5:** Miscellaneous SLC and SLCO transporters regulated by miRNAs.

Transporters	Substrates	miRNAs	Models	Observations	References
SLC4A4/NBCe1	Sodium bicarbonate	miR-224-5p	Colon cancer cell line HT29	Transfection with anti-miR-224 increased SLC4A4 by almost 5-fold	Mencia et al. (2011)
		miR-224-5p	Enamel mineralization Human samples collected from 15- to 25-week-old fetal cadavers	Twofold increase in ameloblast lineage cells SLC4A4 after transfected with miR-224 inhibitor. About 3.5-fold increase in oral buccal mucosal epithelial cells after transfected with miR-224 inhibitor	Fan et al. (2015)
		miR-223-3p	Clear cell renal cell carcinoma cell lines 786-O, ACHN, Caki-1, kidney cell line HK2 TCGA-KIRC dataset, 24 patient samples Pancreatic cancer cell lines AsPC-1, PANC-1, COLO357, HPDE, and prostate cancer PC-3 cells Gene Expression Omnibus database, GSE79634, GSE41369, GSE91035, and GSE16515 datasets 67 paired pancreatic cancer and normal tissue samples Xenograft mouse model	SLC4A4 was decreased by about 50% with miR-223-3p mimic transfection and increased 2–3-fold with miR-223-3p inhibitor	Xiao et al. (2019)
				Transfection with miR-223-3p mimic reduced SLC4A4 mRNA by about 50%, while transection with miR-223-3p inhibitor increased SLC4A4 mRNA by about 2.5-fold	Zhang et al. (2020)
SLC6A1/GAT1	γ-Aminobutyric acid	miR-200c-3p	Clear cell renal cell carcinoma cell line 786-O 82 paired clear cell renal cell carcinoma and normal tissue samples Orthotopic transplantation into mice	Negative correlation between miR-200c-3p and SLC6A1 mRNA	Maolakuerban et al. (2018)
SLC6A2/NET	Norepinephrine Epinephrine Dopamine	miR-579-3p	Samples from unrelated German panic disorder patients HEK293 cell line	Luciferase reporter assay used to confirm the direct regulation of SLC6A2 by miR-579-3p	Hommers et al. (2018)
SLC6A3/DAT	Dopamine	miR-137-3p miR-491-5p	Neuroblastoma cell lines SK-N-SH, SK-N-BE (2), and hepatoma cell line HepG2	Transfection with miR-137 or miR-491 mimic reduced SLC6A3 mRNA by about 60%	Jia et al. (2016)
SLC6A4/SERT	Serotonin	Mmu-miR-16-5p	Depression and anxiety 1C11 murine cells and Hela cells male Swiss-Kunming mice	miR-16 reduced [3H]-paroxetine and [125I]-RTI-55 binding sites specific to SLC6A4 by 40%. SSRI fluoxetine (Prozac) upregulates miR-16 to decrease SLC6A4	Baudry et al. (2010)
SLC6A6/TauT	Taurine γ-Aminobutyric acid	miR-3156-3p	HPV-positive cervical cancer cell lines HeLa, CaSki, SiHa and the HPV-negative CC cells C33A and HT-3 cell lines HPV16/18-positive cervical cancer, HPV-negative cervical cancer, and HPV16/18-negative normal cervical samples	Negative correlation of miR-3156-3p and SLC6A6 protein, but not mRNA	Xia et al. (2017)
SLC9A9	Sodium protons	let-7f-5p	Colitis-associated cancer Colorectal adenocarcinoma cell line Caco-2 Male or female C57BL/6 mice	Transfection of let-7f-5p precursor reduced SLC9A9	Chen et al. (2019)
SLC23A1/SCVT1	L-/D-ascorbic acid Dehydroascorbic acid	miR-103a-3p	Vitamin C transport in intestinal epithelial cells Colorectal adenocarcinoma cell line Caco-2 and colon cell line NCM460 C57BL/6 J male mice	Transfection with miR-103a-3p mimic decreased SLC23A1 mRNA by about 40% and protein by about 30%	Subramanian et al. (2019)
SLC23A2/SVCT2	L-/D-ascorbic acid Dehydroascorbic acid	miR-141-3p miR-200a-3p	Mouse bone marrow stromal cells	Transfection with miR-141-3p inhibitor increased SLC23A2 mRNA by about 2-fold. Transfection with miR-200a-3p inhibitor increased SLC23A2 mRNA by about 50%	Sangani et al. (2015)
DRA/SLC26A3	Chloride	miR-494-3p	Colorectal adenocarcinoma cell line Caco-2 and T-84 cells	Transfection with miR-494 mimic does not downregulate SLC26A3 mRNA but does decrease SLC26A3 protein by 50%–60%	Anbazhagan et al. (2014)
PAT1, SLC26A6	Chloride	miR-125a-5p	Colon adenocarcinoma cell line Caco-2, HT-29, T-84, and SK-CO15 cell lines	Transfection with miR-125a-5p reduced SLC26A6 mRNA by about 49% and protein by 44%	Anbazhagan et al. (2019)
SLC30A8/ZnT8	Zinc ion	miR-143-3p	Glioblastoma cell lines T98G, U87-MG (U87), and A-172 19 clinical glioblastoma samples and five control samples Male and female BALB/c nude mice	Overexpression of miR-143-3p significantly reduces SLC30A8 protein, while miR-143-3p inhibition upregulates SLC30A8 protein	Lozada-Delgado et al. (2018)
SLC34A2/NaPi-IIb	Phosphate	miR-939-5p	Gastric cancer cell lines AGS, BGC-823, HGC-27, MGC-803, MNK-45, SGC-7901, and GES-1 cell lines paired gastric cancer and normal tissue samples xenograft mouse model	Transfection with miR-939 mimic downregulated SLC34A2 mRNA by about 50%	Zhang et al. (2017)
SLC39A6	Zinc	miR-192-5p	Hepatocellular carcinoma cell lines Huh-7, SK-Hep-1, SNU-449, MHCC-97L, MHCC-97H HCC-LM3, kidney cell line HEK-293T clinical paired hepatocellular carcinoma and normal tissue samples Male BALB/c mice	miR-192 overexpression downregulated SLC39A6 protein and direct regulation was confirmed by luciferase reporter assay	Lian et al. (2016)
SLC52A3/RFVT3	Riboflavin	miR-423-5p	Intestinal absorption of riboflavin Colon adenocarcinoma cell line Caco-2 and small intestine adenocarcinoma cell line HuTu-80 male C57BL/6 mice	Transfection with miR-423-5p mimic did not affect SLC52A3 mRNA level but decreased protein by about 50%	Lakhan et al. (2017)

## MicroRNA-Based Therapies

With improved understanding of miRNA biology and regulatory approval of small interfering RNA (siRNA) medications (Yu A.-M. et al., 2020; Moumné et al., 2022; Yu and Tu, 2022), there is great interest in the development of miRNA-based therapeutics. The first siRNA medication, patisiran, was approved by the United States (US) Food and Drug Administration (FDA) in 2018 for the treatment of transthyretin amyloidosis by targeting the 3′UTR of the transthyretin mRNA (Setten et al., 2019). The second siRNA drug, givosiran, was approved by the FDA in 2019 for the treatment of acute intermittent porphyria (De Paula Brandão et al., 2020). Lumasiran was approved by the FDA in 2020 for the treatment of primary hyperoxaluria type 1 by targeting the 3′UTR of hydroxyacid oxidase 1 mRNA (Hulton, 2021). Inclisiran was approved in December 2021 for the treatment of heterozygous familial hypercholesterolemia or clinical atherosclerotic cardiovascular disease by reducing low-density lipoprotein cholesterol *via* the regulation of proprotein convertase subtilisin–kexin type 9, in combination with statin therapy (Raal et al., 2020). It is noteworthy that all of the four siRNA drugs approved by the FDA act on hepatic targets, and among them, three (patisiran, lumasiran, and inclisiran) follow the miRNA mechanism of action to target the 3′UTR (Zhang M. M. et al., 2021; Yu and Tu, 2022). These drugs have proved the concept of RNA interference (RNAi) therapy, including genome-derived miRNAs as therapeutics or targets (Yu A.-M. et al., 2020).

Depending on the miRNA function and dysregulation profile, strategies may employ to either inhibit or restore the miRNA expression or function (Hanna et al., 2019; Yu A.-M. et al., 2020). To inhibit miRNA function, one may use miRNA inhibitors or antagomirs, which are antisense oligonucleotides that bind to miRNA to prevent miRNA from repressing their targets and miRNA competitors, or block-mirs, which prevent the recognition of miRNA binding sites on target mRNA (Setten et al., 2019). There is also a growing interest in developing small-molecule miRNA inhibitors (Ursu et al., 2020; Yu A.-M. et al., 2020; Fu et al., 2021). On the other hand, chemically synthesized miRNA mimics are commonly used to restore miRNA functions in cells. An alternative approach to miRNA mimics has been developed to produce bioengineered RNA molecules (Chen Q.-X. et al., 2015; Ho et al., 2018; Li P.-C. et al., 2018; Deng et al., 2021; Li P.-C. et al., 2021; Tu et al., 2021). The challenges of developing miRNA therapeutics have been discussed in recent articles (Segal and Slack, 2020; Kara et al., 2022; Yu and Tu, 2022), which include minimizing the degradation from nucleases, improving target cell uptake, and avoiding off-target or unwanted side effects.

MRX34, a miR-34a mimic that is effective to suppress tumor progression in animal models through multiple mechanisms including the interference with cancer metabolism via regulating SLC2A1 expression (He et al., 2019; Hong M. et al., 2020), was the first anticancer miRNA to reach phase I clinical trials, but the trial ended in 2016 due to the occurrence of severe immune-related adverse events and even mortality (Hong D. S. et al., 2020). While this trial demonstrates the need for cancer cell-targeted delivery systems since the liposomal nanoparticle SMARTICLE used in the clinical trial is not a cancer cell-specific delivery system (Li W. et al., 2021), caution is advised to select the right molecular entity at the right dose and administer at the right time to achieve efficacious and safe therapy. Nevertheless, there are multiple other therapeutic miRNAs under phase II clinical trials, such as the miR-126 mimic TargomiR and anti-miR-155 Cobomarsen (Kara et al., 2022). Among them, the interactions between tumor-suppressive miRNAs (e.g., miR-126) and SLC transporters (e.g., SLC7A5) not only support the concept of targeting critical SLC transporters for the control of diseases (e.g., cancer) (Zhang et al., 2019; Wang et al., 2020) but also develop effective miRNA medications that may act on multiple therapeutic targets including SLC transporters.

## Conclusion and Perspectives

Our understanding of nutrient metabolism has rapidly developed since Otto Warburg observed aerobic glycolysis in cancer cells in the 1920s. Metabolic reprogramming has been recognized as a hallmark of cancer, and there is ongoing interest in identifying the determinant factors contributing to cancer metabolism, as well as developing respective therapeutic strategies. SLC transporters are commonly dysregulated to support the increased demand for nutrients and metabolites. While some SLCs may not be regulated at the posttranscriptional levels, many studies have demonstrated the direct regulation of SLCs by miRNAs, offering insights into the causes of the altered expression of SLCs in cancer cells. As SLCs are known to transport many critical nutrients as well as endobiotic metabolites, the impact of miRNA–SLC signaling on cancer metabolism cannot be underestimated. Therefore, the intervention of critical miRNA–SLC pathways underlying dysregulated cell metabolism represents a new strategy to control related diseases, including cancer, which may be witnessed in future clinical investigations or even practice. Furthermore, less is known regarding the potential roles of miRNA regulation on drug-transporting SLCs in pharmacotherapy, including possible effects on drug exposure, efficacy, and safety. Therefore, more research in these areas is expected to improve our knowledge on miRNA–SLC interactions in drug development and clinical therapy. Additionally, there are still many orphan SLCs with unknown functions and substrates. Both miRNA and SLCs have a multitude of members, with the well-known receiving a disproportionate amount of attention. It would be beneficial to invest in researching orphan transporters to uncover new interactions and importance in cell metabolism as well as implication to diseases.

With an increased understanding in how some miRNAs regulate cancer-related SLCs, new therapeutic approaches may be developed to either restore or inhibit miRNA functions to control tumor progression and metastasis. Multiple miRNA-based anticancer therapeutics have entered clinical trials. However, the failure of MRX34 due to severe and even fatal adverse reactions highlights the needs for selecting the right therapeutic molecules as well as delivery systems to achieve the desired efficacy and safety. Furthermore, a single miRNA may have multiple targets in the cells, so the full effects of therapeutic miRNA should be carefully defined and considered. Some miRNAs clearly have crucial roles in the regulation of cancer progression, in part, through the direct regulation of key SLC transporters, and the development of miRNA-based therapies, monotherapy or combination with other means, has the potential to vastly improve cancer treatments.

## Abbreviations

AA, amino acid; circRNA, circular RNA; GLUT, glucose transporter; MCT, monocarboxylate transporters; MRE, microRNA response element; miRNA or miR, MicroRNA; ncRNA, noncoding RNA; PPP, pentose phosphate pathway; SLC, solute carrier; 3′UTR, 3′-untranslated region.
